# A Lecture on Albuminuria as a Complication of Pregnancy

**Published:** 1904-11-05

**Authors:** Robert Jardine

**Affiliations:** Professor of Midwifery in St. Mungo's College, Glasgow; Senior Physician to the Glasgow Maternity Hospital


### Hospital Clinics.

### A LECTURE ON ALBUMINURIA AS A COMPLICATION OF PREGNANCY.

By Robert Jardine, M.D., F.R.S.E., Professor of Midwifery in St. Mungo's College, Glasgow; Senior Physician to the Glasgow Maternity Hospital.

I wish to-day to draw your attention to a subject of very great importance, viz., albuminuria as a complication of pregnancy. Normal urine does not contain albumen, and its presence indicates, in most cases, that there is disease of the kidneys. I am referring to cases not connected with pregnancy. When we come to deal with pregnant women we find quite a number of cases show more or less albumen in the urine. Albumen is specially prone to occur in the urine of primiparæ towards the end of pregnancy. In the majority of these cases the kidneys are not diseased in the ordinary sense; there certainly are slight changes in the organs, but one can hardly term them diseased. This condition is known as the "kidney of pregnancy."

In pregnancy an extra strain is thrown upon the kidneys in getting rid of the waste products of the fœtus, as well as of the ordinary waste products of the woman herself. In the majority of cases the kidneys are equal to this extra work, but in some cases they find the strain too great, and the result is albuminuria. Now whenever you find albumen in the urine of a pregnant woman you should regard it as a danger-signal not to be disregarded, as you value the safety of your patient. Albumen in the urine is an infallible sign that the kidneys are not acting properly. Very soon you will find that there is a decrease in the amount of urine excreted, swelling of the feet and ankles soon follows, and in a little while you have dropsy of the arms, face, and body, while in bad cases the labia swell tremendously. Finally, the patient begins to suffer from headache, dimness of vision, and epigastric pain, and convulsions begin. The case is now one of eclampsia, to be discussed in a future lecture. I do not mean to say that every case of albuminuria of pregnancy ends in eclampsia, but I wish to impress you with the fact that some of them do, and that the more albumen there is in the urine the more likelihood there is of eclampsia coming on. A mere trace of albumen may mean nothing, but you must bear in mind that what is a mere trace, say about mid-term, may become a large quantity later on; in fact, what is a trace to-day may be a heavy cloud to-morrow. Albumen in the urine of pregnant women must be regarded as a grave indication of danger, and the larger the amount the graver is the indication of danger. It is a sure sign that the kidneys are not working properly, and that poisonous substances are being retained—poisons of such a nature that the patient may be killed by them.

I have already called your attention to the fact that albumen is found much more frequently in the urine of primiparæ than in that of multiparæ. It is more frequent in young and in elderly primiparæ than in those of an average age—say of 23 to 25. Several of our recent cases have been elderly multiparæ, and I have seen quite a number of such cases, so that I believe it more likely to occur among them, than in multiparæ of, say, 35.

I have told you that albumen in the urine does not necessarily indicate kidney disease, but if a woman has had kidney disease prior to conception albumen will be sure to appear. We must never lose sight of the fact that kidney disease may be present. This is especially true of cases occurring in multiparæ. In such cases the danger is greatly increased. It is therefore of importance to ascertain if the patient has had anything wrong with her kidneys previously. If she has had scarlet fever within recent years it is probable her kidneys have been damaged, for inflammation of the kidneys is a very common complication of scarlet fever.

### Diagnosis of Albuminuria.

If a patient is dropsical, you should at once suspect albuminuria and have the urine tested. There are various tests for albumen, but I shall only mention one, viz., heat. If you boil a specimen of albuminous urine in a test-tube or in an iron spoon, a white cloud will form, varying in intensity according to the amount of albumen present. If phosphates are present in the urine, a slight white cloud will form on boiling the urine, and you may be misled by this; but if you add a few drops of dilute acetic acid or vinegar, the cloud of phosphates will at once disappear, while the cloud of albumen will not. It is usual to add two or three drops of acetic acid to the urine before boiling it to prevent this deposit of phosphates. The amount of albumen varies greatly: in some urines it may be only a haze; in others a heavy cloud; in others, again, it may become nearly solid; while in a few it actually becomes so solid that you can invert the test tube without spilling the contents.

Œdema of the feet and ankles does not always mean that there is albumen in the urine. The swelling is often caused by the pressure of the gravid uterus upon the large veins, impeding the circulation; but it is a safe rule to examine the urine in every case in which there is the least swelling of these parts. Indeed, if at all possible, one should examine the urine of every pregnant woman under one's care several times in the later months of pregnancy. With better-class patients this is possible, but it is very difficult among the working classes. If I cannot get specimens of urine to examine, I always warn my patients to let me know at once if they find their feet beginning to swell. I am specially careful to impress upon primiparæ the importance of this measure. The following case well illustrates the importance of carefully watching for albumen in the urine of primiparæ. Last summer I was engaged to attend a primipara, and told her I wished to have specimens of her urine for examination. As the lady was a foreigner, and could speak very little English, I had to make my request through the husband. He at once said, "You want to test for albumen. I can do that for you. In fact, I have been doing it frequently since my wife became pregnant." He continued the testing each week, and about six weeks before full time reported its sudden appearance. I at once put the patient under treatment, and kept her under treatment until she was delivered. In that case I am sure that, without careful supervision, the result would have been disastrous. Even as it was, I was not easy in my mind until the patient was safely delivered.

In bad cardiac cases there will be marked dropsy, and albumen will be found in the urine. Such cases, however, will show indications of heart disease, such as breathlessness, palpitation, cough, and blueness of the face.

*Prognosis.*—If there is a large quantity of albumen and the amount of urine excreted is small the condition is a very grave one, and there is immediate danger of eclampsia. If there is only a small quantity of albumen present, and if urine is being passed in large quantities, the immediate risk is not great; but the patient must be carefully treated, because the amount of albumen may suddenly increase at any time, and the excretion of urine may diminish. A chill or any indiscretion in diet may bring this about.

*Treatment.*—The treatment adopted should aim at relieving the patient's system of the poisonous substances which are accumulating. The bowels should be moved very freely by means of a purge which will cause a copious watery evacuation. Jalap may be used but Epsom salts is the one we chiefly use. It may be necessary to give a saline purge each morning, as at least one free evacuation must be obtained each day. The kidneys should be acted upon by diuretic medicines. The patient should be encouraged to drink large quantities of Imperial drink, *i.e.*, a cream of tartar drink. The more fluid she takes, the better the kidneys will be flushed out. The patient should be carefully guarded against chills, and in bad cases should be confined to bed. A hot bath will cause the skin to act well and will relieve the system wonderfully. In bad cases we invariably give a hot pack. The dieting of the patient is of the greatest importance. All meat should be forbidden, and nothing but milk allowed. A strict milk diet is the one for such patients, and, so long as there is albumen in the urine, they must not be allowed to depart from it. If they take any butcher meat, an increase in the amount of albumen almost invariably occurs within the next 24 hours.

In most cases the albumen will disappear if the patient is carefully treated, but, if the case is one of real kidney disease, the albumen will not entirely disappear. During labour it will probably reappear or increase in amount. After labour it will entirely disappear in a day or two if the case is one of "the kidney of pregnancy," but if it is a case of kidney disease it will persist, although it will lessen much in amount. If during the puerperium the patient should become fevered, the albumen will reappear in large quantities.

